# Pedicled transfer of the clavicular head of the pectoralis major muscle for deltoid deficiency in reverse total shoulder arthroplasty

**DOI:** 10.1016/j.xrrt.2026.100798

**Published:** 2026-06-12

**Authors:** Jose Carlos Garcia, Ricardo Berriel Mendes, Jesely Myrrha Garcia, Paulo Cavalcante Muzy

**Affiliations:** a NAEON Institute, São Paulo, São Paulo, Brazil; b Moriah Hospital, São Paulo, São Paulo, Brazil; c Vila Nova Star Hospital, São Paulo, São Paulo, Brazil

**Keywords:** Tendon transfers, Pectoralis muscles, Deltoid muscle, Nerve injuries, Axillary nerve, Reverse total shoulder arthroplasty

Reverse total shoulder arthroplasty (rTSA) has consistently demonstrated excellent functional outcomes in patients with preserved deltoid function.^1^ However, partial or complete deltoid paralysis remains a challenging clinical condition, as the absence of a functional deltoid compromises both active elevation and prosthetic stability.^2^ In such cases, rTSA alone is often insufficient to restore meaningful shoulder function, and adjunctive procedures must be considered.

For patients with significant deltoid impairment who are candidates for rTSA, pedicled transfer of the entire pectoralis major muscle has been proposed as a strategy to restore active elevation, given its strength and excursion.^3^^,^^4^

Clinical reports have demonstrated that this transfer can provide a degree of active forward elevation and functional improvement when combined with rTSA.^3^ Nevertheless, this approach is associated with important drawbacks, including cosmetic deformity, extensive scarring, and a nonintuitive rehabilitation process, as patients must relearn shoulder elevation using a muscle with a different primary function.

From a neurophysiological perspective, transferring the entire pectoralis major may not be optimal. The sternal head is predominantly innervated by the medial pectoral nerve, receiving contributions from the C7, C8, and T1 nerve roots, which are not primarily associated with deltoid function. In contrast, the clavicular head is mainly innervated by the lateral pectoral nerve, with contributions from the C5, C6, and often C7 roots,^7^^,^^8^ corresponding to the same roots responsible for deltoid activation. This anatomical and neurophysiological alignment can suggest that selective transfer of the clavicular head of the pectoralis major (CHPM) may allow for a more synergistic and intuitive motor recruitment pattern.

In this context, transferring only the superior (clavicular) portion of the pectoralis major for deltoid palsy was proposed^5^; however, these patients were not candidates for rTSA. Building upon this concept, a more targeted transfer may reduce donor site morbidity while preserving the function of the remaining muscle.

The purpose of the present technical note is to describe a pedicled transfer of the CHPM performed in conjunction with rTSA in 5 patients with deltoid paralysis.

## Author's indication and contraindication

### Indications

All patients met criteria for primary or revision reverse shoulder arthroplasty plus at least one of the following:

1. Irreversible anterior or complete deltoid paralysis secondary to axillary nerve lesion, with no anticipated spontaneous recovery and no option for nerve repair or reconstruction.
2. Confirmation of advanced deltoid atrophy and/or fatty degeneration on magnetic resonance imaging or ultrasound.
3. Contraindication to nerve transfer or prior failure of nerve transfer procedures.

### Contraindications

1. Previous surgery, injury, or disease involving the pectoralis major muscle that impairs its viability or range of motion.
2. Extensive scarring or soft-tissue contracture in the anterior chest or shoulder precluding safe pedicled flap elevation.
3. Neurologic injury to the upper trunk, lateral cord, or lateral pectoral nerve causing severe atrophy or malfunctioning of the CHPM muscle.
4. Significant medical comorbidity or poor patient compliance likely to increase perioperative risk or compromise post-operative rehabilitation.
5. Active infection in the operative field or adjacent soft tissues.

### Surgical procedure

The patient is positioned in the beach-chair position under general anesthesia supplemented by an interscalene block. A shoulder chair and arm positioner facilitate limb placement.

**Figure 1:**
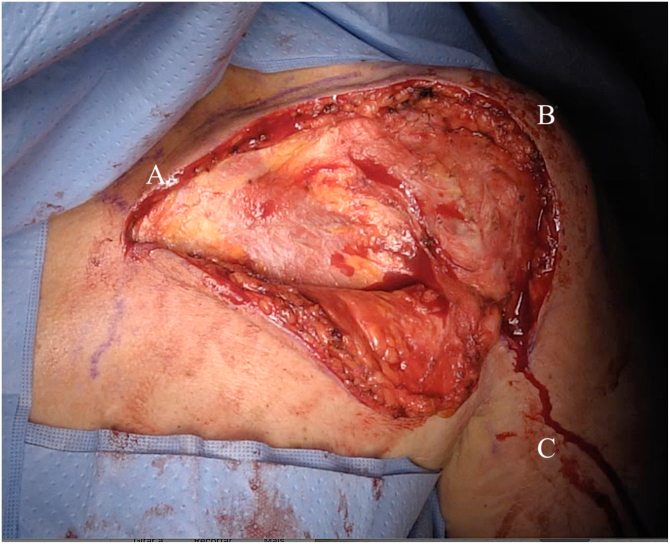
Image of the approach for rTSA with transfer of the CHPM. (A) Sternoclavicular joint. (B) Shoulder. (C) Arm. *rTSA*, reverse total shoulder arthroplasty; *CHPM*, clavicular head of the pectoralis major.

A vertical incision starts at the distal pectoralis major humeral insertion, extends proximally along the humerus to the anterior portion of the lateral acromion, then curves medially along the anterior acromion and clavicle to the sternoclavicular joint, and finally descends 2 cm parallel to the sternum (Fig. 1).

Dissection exposes the cephalic vein; the anterior and some lateral deltoid fibers are detached as needed.

**Figure 2:**
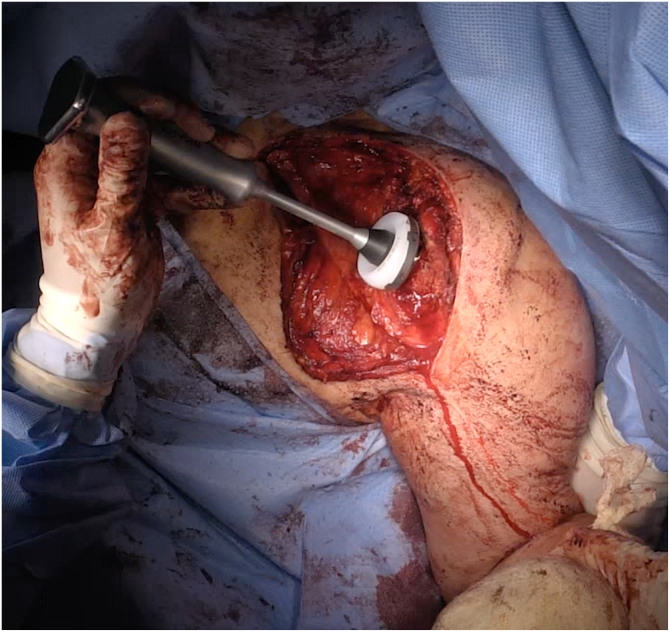
Insertion of the rTSA. *rTSA*, reverse total shoulder arthroplasty.

The CHPM is identified. Standard rTSA implantation follows, using a lateralized design, that lateralizes and inferiorizes the original CHPM insertion site, eliminating the need for tendon detachment from the humerus (Fig. 2).

**Figure 3:**
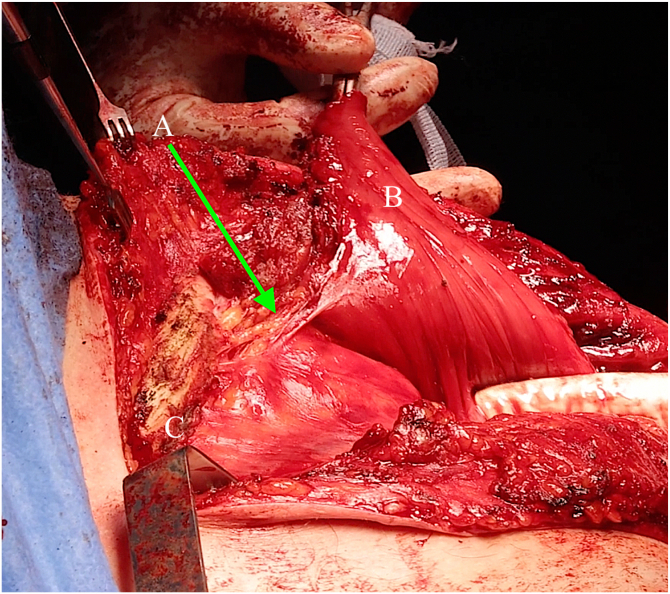
(A) Neurovascular pedicle to the CHPM. (B) CHPM as flipped book sheet. (C) Proximal clavicle near the sternoclavicular joint. *CHPM*, clavicular head of the pectoralis major.

The CHPM origin is released from medial to lateral with electrocautery, leaving a small 1-2 cm lateral bridge intact to protect the neurovascular pedicle and guide optimal tensioning. The pedicle, usually situated near the middle third of the clavicle, is carefully preserved during mobilization (Fig. 3).

Once sufficiently mobilized for appropriate tension, the muscle is turned laterally like opening a book page.

**Figure 4:**
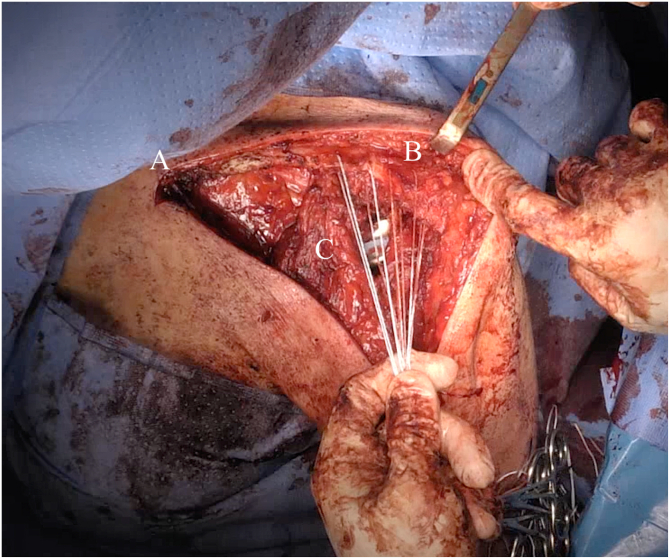
(A) sternoclavicular joint. (B) Anterolateral acromion. (C) Released CHPM leaving a small 1-2 cm lateral bridge intact. *CHPM*, clavicular head of the pectoralis major.

The recipient bone is prepared by decorticating the lateral clavicle and anterior/anterolateral acromion with a burr to bleeding cancellous bone. Transosseous fixation employs high-strength #2 nonabsorbable sutures passed from clavicle to lateral acromion (Fig. 4).

Muscle perfusion and contractility are confirmed throughout by tissue color and response to gentle needle stimulation. The shoulder is held in neutral rotation and 60° forward flexion during fixation to ensure proper length and alignment.

**Figure 5:**
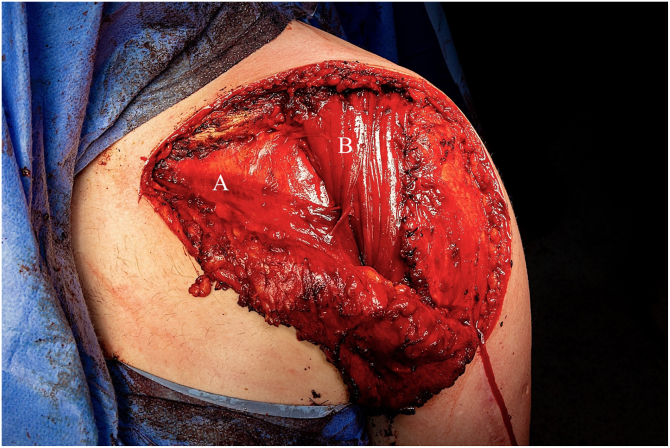
(A) Native clavicular footprint and thoracic apposition area of the CHPM. (B) The new clavicular and acromial footprints and apposition area of the CHPM over the rTSA. *rTSA*, reverse total shoulder arthroplasty; *CHPM*, clavicular head of the pectoralis major.

The CHPM tendon is sutured to the residual deltoid and its native insertion site to enhance tension distribution and load sharing. Other absorbable sutures approximate the atrophic but preserved native deltoid to the transferred CHPM, maintaining anatomic continuity despite deltoid malfunction (Fig. 5) (Supplementary Video).

### Post-operative rehabilitation protocol

The shoulder remains immobilized in an abduction sling set at 45° for 6 weeks. Passive motion is prohibited to protect muscle-bone healing.

During brace removal for dressing or bathing, patients must keep the shoulder at 45° forward flexion.

From week 6: gentle active-assisted range-of-motion exercises begin in gravity-eliminated position, avoiding movements against the transfer direction. Light resistance and simple low-demand functional activities are started.

From week 8: progressive stretching for range of motion. Patients gradually resume daily activities with the operative arm, advancing based on comfort, strength, and functional progress.

### Clinical cases

All the 5 patients assessed in this study (2 females and 3 males) demonstrated improvement in active forward flexion, with a mean of 75° (range, 60°-90°), and in abduction, with a mean of 47° (range, 30°-65°). Passive range of motion was not assessed.

Three patients had undergone at least 3 previous procedures each before referral to our service. All 3 required revision of the previous shoulder arthroplasty.

The poorest outcome occurred in a patient undergoing revision surgery for a periprosthetic fracture around a nonstandard shoulder endoprosthesis associated with complete axillary nerve injury. In this case, we performed a cemented revision rTSA with bone allograft reconstruction and plate fixation, combined with soft-tissue reattachment to the allograft and CHPM transfer. Forward flexion remained the lowest in the cohort, at 60°.

The other 2 revision cases were treated with CHPM transfer combined with noncemented rTSA. These procedures were revisions for 1 failed shoulder cemented hemiarthroplasty and 1 failed cemented rTSA, both with compromise of the anterior and lateral deltoid muscles. These patients achieved forward flexion of 110° and 95°, respectively.

The remaining 2 cases were not revisions of prior arthroplasties. Both patients had previously undergone surgery for proximal humeral fractures and subsequently developed post-traumatic osteoarthritis. They were also referred with axillary nerve injury caused by improper anterolateral surgical approaches, resulting in atrophy of the anterior and part of the lateral deltoid muscle. In both cases, treatment consisted of CHPM transfer combined with noncemented rTSA.

Post-operative forward elevation was 145° and 120° in these 2 cases. Angular measurements were obtained using manual goniometry in 5° increments.

No patients were excluded from the study for any reason.

Patient data are presented in Table I.

**Table I:** Data of the patients.

Patient	FF before	FF after	Ab before	Ab after	Age	FU	Side	Revision	Graft
1	0°	60°	0°	30°	62	98	R	Y	Y
2	35°	120°	25°	75°	41	55	R	N	N
3	70°	145°	60°	100°	60	43	L	N	N
4	20°	110°	15°	80°	66	13	L	Y	N
5	30°	95°	30°	80°	35	12	L	Y	N

*FF*, active forward flexion; *Ab*, active abduction; *FU*, follow-up.

## Discussion

rTSA has demonstrated reliable pain relief and functional improvement in cuff-deficient shoulders when deltoid function is preserved.^2^ However, deltoid paralysis remains a major challenge because rTSA depends on a functional deltoid to generate active elevation and maintain prosthetic stability.^4^ In this setting, rTSA alone is frequently insufficient, and adjunctive reconstructive procedures may be required.

The principal finding of this technical note is that pedicled transfer of the CHPM, performed in conjunction with rTSA, was associated with improved forward flexion in all 5 patients. The degree of recovery varied according to case complexity. The best results were observed in the 2 nonarthroplasty revision cases, both of whom had previous osteosynthesis for proximal humeral fracture complicated by axillary nerve injury with anterior and part of the lateral deltoid affected. Among the 3 revision arthroplasty cases, forward flexion reached 110°, 95°, and 60°. The poorest result occurred in the most severe salvage situation, involving periprosthetic fracture, complete axillary nerve injury, major bone reconstruction, and prior instability. Even in that case, however, instability resolved after rerevision with implant optimization and CHPM transfer.

These findings support prior reports suggesting that muscle transfer may expand the indications for rTSA in patients with deltoid deficiency. Studies have emphasized the difficulty of performing rTSA in deltoid palsy,^2^ a condition historically considered a major limitation, to rTSA^4^ Other studies subsequently demonstrated that rTSA combined with pedicled transfer of the pectoralis major can restore a useful degree of active elevation in this patient population.^3^^,^^4^ The present technical note supports this concept while proposing a more selective transfer, limited to the clavicular head rather than the entire muscle.

Selective transfer of the CHPM may offer relevant mechanical and biological advantages. From a biomechanical standpoint, the clavicular head has a line of pull more closely aligned with forward flexion and anterior elevation, making it a logical substitute for anterior deltoid deficiency A study that electromyographically assessed the different regions of the deltoid 2 years after rTSA suggested that the posterior deltoid does not effectively contribute to shoulder flexion, abduction, or extension.^6^ These findings suggest that the anterior and lateral deltoids are more closely associated with long-term shoulder movement after rTSA. Therefore, isolated transfer of the CHPM seems rational, as it may replace the function of the anterior deltoid and even part of the middle deltoid.

By contrast, transfer of the entire pectoralis major may provide a less specific force vector and can be associated with greater donor-site morbidity, cosmetic deformity, and loss of residual native muscle function.

Preserving the sternal head may in contrast reduce morbidity while maintaining part of the normal function and contour of the anterior chest wall.

There is also a neurophysiological rationale for this selective transfer. The clavicular head is predominantly innervated by the lateral pectoral nerve, with contributions mainly from C5-C6 and often C7, overlapping the roots primarily responsible for deltoid activation through the axillary nerve. In contrast, the sternal head is more strongly influenced by the medial pectoral nerve and lower root levels^7^^,^^8^ This segmental similarity suggests that transfer of the CHPM may allow a more synergistic and intuitive recruitment pattern than transfer of the entire muscle. Although this hypothesis cannot be confirmed by the present technical note, it is consistent with the reconstructive concept proposed by a study who described transfer of the superior portion of the pectoralis major for restoration of shoulder abduction in patients with deltoid palsy outside the rTSA setting.^5^

An additional technical advantage of this procedure is that CHPM provides a similar length to the anterior and part of the lateral deltoid. This advantage, combined with the lateralized rTSA design, allows preservation of the native humeral insertion of the CHPM. By effectively lateralizing and distalizing the reconstructed shoulder, the prosthetic construct facilitated CHPM transfer tensioning without requiring tendon detachment from the humerus. This may simplify the procedure and preserve muscle biology. In addition, suturing the residual deltoid to CHPM and maintenance of CHPM native insertion, may improve load sharing and force transmission.

The variability in post-operative motion observed in this technical note likely reflects the reconstructive environment rather than the transfer alone. Patients with less severe prior surgical damage and more favorable local tissue conditions achieved greater forward elevation, whereas the most complex salvage case had the lowest final motion. This suggests that CHPM transfer should be viewed as an adjunct capable of restoring meaningful function, but not necessarily normalizing shoulder performance in highly compromised shoulders. The results achieved in these 5 cases seem to be similar to those reported for techniques of clavicular head associated with sternal head transfer.^3^

The apparent contribution to stability in the most complex revision case is also noteworthy, as restoration of an anterior-superior soft-tissue vector may have improved dynamic centering of the reverse prosthesis.

This study has several limitations. It is a small uncontrolled report with heterogeneous indications and varying degrees of prior surgery, bone loss, and neurologic injury with no functional assessments unless forward flexion.

Despite these limitations, this technical note introduces a technically feasible and biologically rational modification of previously described pectoralis transfer strategies for patients with deltoid dysfunction undergoing rTSA.

## Conclusion

Pedicled transfer of the CHPM performed in conjunction with rTSA may be a useful option for selected patients with anterior or complete deltoid dysfunction; however, a case series is needed to better understand benefits and drawbacks of this procedure.

## Disclaimers:

Funding: No funding was disclosed by the authors.

Conflicts of interest: Jose Carlos Garcia Jr is consultant of Zimmer Biomet and Stryker. Given his role as Associate Editor, Jose Carlos Garcia Jr had no involvement in the peer-review of this article and has no access to information regarding its peer-review. Any additional authors, their immediate families, and any research foundations with which they are affiliated have not received any financial payments or other benefits from any commercial entity related to the subject of this article.
